# A theory of social thermoregulation in human primates

**DOI:** 10.3389/fpsyg.2015.00464

**Published:** 2015-04-21

**Authors:** Hans IJzerman, James A. Coan, Fieke M. A. Wagemans, Marjolein A. Missler, Ilja van Beest, Siegwart Lindenberg, Mattie Tops

**Affiliations:** ^1^Department of Clinical Psychology, VU University, Amsterdam, Netherlands; ^2^Department of Psychology, University of Virginia, Charlottesville, VA, USA; ^3^Department of Social Psychology, Tilburg University, Tilburg, Netherlands; ^4^Department of Developmental Psychology, Tilburg University, Tilburg, Netherlands; ^5^Department of Sociology, University of Groningen, Groningen, Netherlands

**Keywords:** social thermoregulation, embodiment, social cognition, attachment theory, development, neural reuse, economy of action

## Abstract

Beyond breathing, the regulation of body temperature—*thermoregulation*—is one of the most pressing concerns for many animals. A dysregulated body temperature has dire consequences for survival and development. Despite the high frequency of social thermoregulation occurring across many species, little is known about the role of social thermoregulation in human (social) psychological functioning. We outline a theory of social thermoregulation and reconsider earlier research on people’s expectations of their social world (i.e., attachment) and their prediction of the social world. We provide support and outline a research agenda that includes consequences for individual variation in self-regulatory strategies and capabilities. In our paper, we discuss physiological, neural, and social processes surrounding thermoregulation. Emphasizing social thermoregulation in particular, we appeal to the economy of action principle and the hierarchical organization of human thermoregulatory systems. We close with future directions of a crucial aspect of human functioning: the social regulation of body temperature.

## Introduction

Beyond breathing, *thermoregulation*—the regulation of body temperature—is the most immediate problem for many animals. Food can be stored and budgeted. Mating depends on survival. But survival depends first on oxygen and an adequately heated (or cooled) body. Indeed, a dysregulated body temperature has dire consequences. Normal development in infants requires an optimal internal body temperature across a wide variety of mammalian species—especially in infants born prematurely. Hibernating animals, which maintain a very low body temperature during hibernation, can die during abnormally long winters (Boyles and Brack, 2009). And elderly humans—whose ability to regulate body temperature is often compromised (Van Someren et al., 2002; Shibasaki et al., 2013)—sometimes die during abnormally hot or cold seasonal variations (Mallet, 2002). Finally, in early development, infants require body heat from the caregiver to stay alive, for some animals even to the detriment of the caregiver, who risks death in exchange (Adels and Leon, 1986; Gilbert et al., 2007).

Across many animal species, animals to seek and maintain *thermoneutrality*—a strategy that can be characterized as seeking temperature-specific homeostasis—by maintaining a gradient between the core temperature and the body’s peripheral temperature (Cannon, 1929; Mallet, 2002).^1^ A striking feature of thermoregulation is its social nature:across many species, fellow animals help to warm in times of danger, disease, and distress, because the net energy cost of thermoregulation decreases when it is done through bodily contact with others. Typically, social thermoregulation occurs within obvious ingroups or among close kin (like mother–child relationships, or between romantic partners), what others have called a *Communal Sharing* relationship (cf. Fiske, 1991, 1992).^2^ In our paper, we summarize how social thermoregulation functions across many animal species, discuss how social thermoregulation functions in the human body and brain (in particular its role in the generalized regulation of bioenergetic resources; q.v. Beckes and Coan, 2011), and consider broader implications for psychological functioning.

What do we mean by social thermoregulation? The most basic function of social thermoregulation is to help warm another agent, either through touch, or through a warming of the skin in the supporter, where the skin-warming could be a mediating effect while touching the agent. These effects occur in many animals, including humans. We think that higher order social cognitions and behaviors—like attachment, social emotional functioning, and affect regulation—are scaffolded on top of these more basic functions. And some empirical support indeed reveals that social thermoregulation has broader consequences for social cognition, such as attachment (mental models of self and others), emotional functioning, and the degree to which people possess the capacity to self-regulate (consciously or not; cf. Holt-Lunstad et al., 2008). We will discuss these higher order social cognitions in more detail at the end of this review.^3^

In our review we use heuristics to understand how parts of the brain may have matured, and how some more novel functions of social cognition and behavior—like providing social support to another—have built up from more plesiomorphic social thermoregulatory functions (Mandler, 1992). That is, we think that social behavior is partially embedded within a thermoregulatory system that (a) includes both reactive and predictive cues (from adjusting to environmental temperatures to giving a sad friend a warm hug), and is (b) hierarchically organized in terms of bioenergetic cost (Satinoff, 1978, 1982; Proffitt, 2006; Beckes and Coan, 2011; Morrison and Nakamura, 2011). More broadly, our contribution is based on four propositions, and we propose a number of different conjectures that we hope will guide research in this area. The first proposition is that much of animal life is not possible without proper thermoregulation. Second, thermoregulation is bioenergetically costly. Third, energy expenditures are diminished by social thermoregulation, when the costs of thermoregulating are shared within social groups or dyads. Fourth, efficient energy conservation through social thermoregulation happens both throughout development and in later life, and both have consequences for the development of physiological mechanisms supporting attachment, affect, perception, and social cognition (Coan et al., 2006; Coan, 2008; IJzerman and Koole, 2011).

The ultimate purpose of our review is to explain basic social thermoregulation and how early social thermoregulation serves the development of higher-level cognition. In order to do so, we discuss what kind of innate expectations humans are likely to have in regards to being social thermoregulated in the early stages of their life, and how these innate mechanisms have come to rely on core features required for the effective regulation of resources. The most prominent innate mechanism (though certainly not the only one) we think is social thermoregulation.^4^ We will also suggest that thermoregulatory development can be altered through caregiving style, in a way that allows a new look at the classical idea of attachment theory.

After discussing the integration of social thermoregulation during development and what this means for people’s predictive models of others, we discuss how self-regulatory processes in humans may be influenced to a considerable degree by thermoregulation and the processes around it. To date, we are not aware of any other thermoregulation-based model of social cognition, and we hope that our comprehensive review contributes to much needed theory formation related to temperature cues. Finally, we think that our theory provides a first explication of how (and why) social thermoregulation contributes to attachment, emotional functioning, and the capacity to self-regulate. In order to track progress regarding our theoretical presumptions, we have created a project page on the Open Science Framework and we encourage colleagues to post studies related to our theory (https://osf.io/7d94r/). We propose social thermoregulation to be a key feature of social life, and many existing findings can be interpreted in light of our framework. We do realize that many links between physiology and cognition still remain informed conjecture, but we set an agenda to investigate these important links.

## Behavioral Thermoregulation and its Physiological and Neural Mechanisms

The relationship literature is replete with studies that approximate a fuller understanding of how people relate to one another. The diversity of these approaches is enormous: They have included cognitive representations of non-close vs. close others through metaphorical “inclusions” of “others into the self” (Aron et al., 1992) or mechanisms that are central to the maintenance of communal sharing relationships through verbal capitalizations (Reis et al., 2010), and they have extended to co-regulatory physiological processes (Sbarra and Hazan, 2008; Waters et al., 2014), and so forth. Our focus predominantly explicates a level lower than most of these approaches, and we focus on the closest of people’s relationships: Communal Sharing relationships.

Many related approaches have thus already focused on lower level processes in service of formation and maintenance of the communal sharing relationships, but we believe we offer a very basic—and novel—regulatory mechanism. We already know a considerable amount regarding the secretion of neuromodulators such as oxytocin and vasopressin in relation to the maintenance of close relations (e.g., Insel and Young, 2001). Case in point is a variety of research on breast-feeding (e.g., Uvnäs-Moberg and Eriksson, 2008) and intercourse (e.g., Carter, 1992). Our approach focuses on a related process that we see—as a perceptual mechanism—crucial to understanding social relations. Bowlby (1969) thought attachment to first rely on “perceptual building bricks,” that are “activated by stimuli falling within one or more broad ranges, [are] terminated by stimuli falling within other broad ranges, and [are] strengthened or weakened by stimuli of yet other kinds” (p. 625; see also IJzerman and Koole, 2011). These perceptual building bricks should first determine children’s expectations, and then afford people to let close others share the risks of living in dangerous (e.g., cold) environments and help carry energetic burdens (e.g., Beckes and Coan, 2011).

But despite Bowlby’s (1969) stress on building bricks (activated through for example touching or clinging), thus far we only have rudimentary understandings of the exact mechanisms that allow almost reflexive responses in Communal Sharing relationships, a feature that has been regarded central to the attachment literature. Our perception-oriented focus on social thermoregulation further allows comprehension of how people *communicate* their intentions to bond, how these intentions affect (early) memories of their social relations, and how people engage in bonding experiences—even when they do not have control over language. Furthermore, these building bricks should play an important role in the formation of the internal model of relationships (cf. Craik, 1943). In all of these, we suggest, social thermoregulation plays an important role. But to understand whether thermoregulation plays a role at all in the higher order social behaviors and cognitions, we need to know how neurological and physiological mechanisms behave in the context of behavioral and social thermoregulation.

### A Framework of Reactive and Predictive Temperature Homeostasis

As a general rule, we presume that thermoregulatory mechanisms in many animals are (physiologically and cognitively) hierarchically organized by bioenergetic costs, which follows from the more general principle of biological and cognitive systems referred to as the “economy of action,” as well as the tendency to re-use more ancient brain mechanisms for more novel purposes (e.g., Anderson, 2010). The economy of action principle manifests as a general tendency toward energy conservation where possible, due to the uncertainty of resources and the need to bring in more resources than are expended (Davies et al., 2012).

Central to the notion of hierarchical organization—and crucial for the principle of economy of action—are generalized imperatives to predict and control one’s environment that should be visible in all regulation mechanisms described here. Challenges to prediction and control trigger a host of neural and physiological responses (Sapolsky, 2005; Clark, 2013). Physiological responses to such challenges take one of two forms: reactive homeostatic responses arise to feedback signaling changes in physiological variables that have already occurred or were not predicted. Predictive homeostatic responses emerge in anticipation of predictably timed challenges (Moore-Ede, 1986; Romero et al., 2009; cf. Landys et al., 2006).

Both basic predictive and reactive homeostatic control mechanisms are foundational to what Tops et al. (2014b) have dubbed predictive and reactive control systems (PARCS). When a challenge or task is perceived as predictable and controllable, *predictive* homeostasis is maintained. By contrast, situational novelty and unpredictability require effortful feedback processing and can trigger *reactive* physiological responses that are subjectively effortful and potentially incur health costs (Romero et al., 2009). In terms of thermoregulation, reactive control (e.g., the experience of psychological stress) increases core body temperature and decreases skin temperature by restricting blood flow to extremities (e.g., Rimm-Kaufman and Kagan, 1996: Porges, 2001). In this way, reactive control serves to avert physical harm. But its implementation comes at an energetic cost.

Besides harm avoidance, energy acquisition and storage are important prerequisites for reproductive success (Bronson, 1989). Thus, in most species, behavioral sequences are organized so that a period of eating and fattening often proceeds mating and caring for offspring. This is particularly important in habitats where food availability fluctuates or is unpredictable (Schneider et al., 2013). In species representing every vertebrate taxa and even in some invertebrates, many putative “satiety” or “hunger” modulators including oxytocin and serotonin, function to schedule ingestive behavior in order to optimize reproductive success in environments where energy availability fluctuates (de Matos Feijó et al., 2011; Schneider et al., 2013). The same modulators such as oxytocin and serotonin are also implicated in thermoregulation. For example, the secretion of oxytocin contributes to vasodilation, a state in which the blood flows to the periphery and leads to an increased skin temperature (Lowry et al., 2009). These substances are also reliably related to a wide variety of social behaviors, with its main function to be motivated to return to social predictability.

In predictable environments, models can be formed that optimize predictive control. Predictive control, in turn, allows energetic expenditure to be *scheduled*, precluding the need for costly reactive control. The activation of reactive or predictive control mechanisms depends on perceptions of threats to physiological (e.g., food) and/or social resources, as well as generalized predictability (cf. Stearns, 1992). We reason that early social experiences not only facilitate the formation of internal predictive models of attachment, but because of this formation it may induce general inclinations toward either reactive or predictive control.

A shift from reactive toward predictive temperature control is particularly important because reactive thermoregulation can be so costly. This shift may be seen as one from reactive thermoregulation for survival toward predictive thermoregulation for care and attachment for sharing the thermoregulatory load (cf. Coan and Sbarra, 2014). In evolutionary terms, the development of more predictable forms of social thermoregulation may have been based on the importance of thermoregulation in the care for offspring. It is likely that parental thermoregulation of offspring evolved initially to reactively control unpredictable and dangerous environments. In line with prevailing theories that mechanisms of adult attachment and affiliation evolved from mechanisms of caregiving or mother-offspring attachment (Van IJzendoorn, 1995), the development of predictive thermoregulatory control via social channels may have involved an expansion and generalization from care for offspring to adult attachment. Increased social thermoregulatory control may in turn have increased the predictability of prevailing environmental circumstances, and with that, possibilities for extended investment of time and resources toward offspring, facilitating *their* predictive model development. Before expanding on this last possibility, we now turn to how temperature control works at different hierarchically nested levels.

### Behavioral Thermoregulation for the Solitary Animal

The most basic component in our hierarchy of thermoregulation is *behavioral thermoregulation*, a way in which many animals exert reactive control to adapt their own temperature, and several types of animals achieve this in different ways (simply in their attempts to stay alive by conserving energy). Related to the notion of predictive and reactive control—and its relation to energy expenditure—an organism’s environmental temperature plays a crucial role in shaping metabolic activity, while it also influences sexual reproductive behaviors across species (Bronson, 1989). But what are some ways in which animals thermoregulate themselves?

The most basic form of thermoregulation can be engaged in by poikilotherms (environmentally dependent, cold-blooded animals), who regulate their temperature using their external environment. For example, wood turtles move from one area to the other (from forest clearings into streams) to use the environmental temperature to regulate their own body temperature (Dubois et al., 2009; Akin, 2011). Homeotherms (animals with constant, environment independent and typically high body temperatures), like poikilotherms, can use their environment to regula their temperature. In addition, they rely on internal physiological mechanisms to regulate body temperature when external temperatures are within non-threatening limits [such as through shivering through the contraction of skeletal muscles, or sweating through the catabolization of brown adipose tissue (BAT); Grigg et al., 2004; Ivanov, 2006]. This way of regulating body temperature allows the skin temperature to drop, so that core body temperature can be conserved (Akin, 2011). Another way of regulating body temperature for nocturnal animals, like skunks, is to enter torpor—a state that is characterized by decreased body temperature and minimizing energy loss—from midnight until dawn (Hwang et al., 2006).^5^

### Neural Mechanisms Underlying Behavioral Thermoregulation

One of the most important tasks of the body outside of maintaining an oxygen supply is the regulation of body temperature. In fact, the range of tolerable temperatures within the brain and bodily core is small (Kurz, 2008). Although the brain in particular is capable of surviving for short periods at relatively low temperatures—a condition followed by generalized torpor and disorientation—even small increases in temperature will cause neuronal dysfunction and death (Burger and Fuhrman, 1964; Cabanac and Caputa, 1979). In order for the organism to survive, it thus needs to be able to cool its own body by itself. Interestingly, unlike the maintenance of a continuous supply of oxygen via the lungs, there is no “temperature” organ in the body, despite the fraught nature of temperature in the struggle to survive. Thus, the brain and body contain multiple, often redundant capacities for predicting and adapting to changes in ambient and core temperature, in order to keep those temperatures within optimal limits.

These capacities range from prefrontal systems tasked with predicting changes in ambient temperature (frequently related to predictive models) to cutaneous effectors capable of communicating ambient temperature information to the central nervous system and thermosensitive cells *within* the central nervous system capable of detecting even small changes in the temperature of circulating blood (Nakayama et al., 1961; Hori, 1991). Planning for future potential temperature shifts, mediated by cortical prefrontal and midline structures, are part of an all-purpose method of maintaining optimal conditions for health and well-being. These processes are capable of contingency planning, prediction and vigilance, and offer the advantage of *precluding* the need for other systems dedicated to reactive thermoregulatory effort (like while watching the weather report and bring an extra coat for use during a chilly day to warm up).

Those on-line methods for detecting internal and external fluctuations in temperature are themselves complex. The mechanisms through which the body responds to challenges to core body temperature are more complex still, and, as we cannot emphasize enough, they are organized hierarchically in terms of their own energy demands. What follows here is a brief overview of these systems and processes. In subsequent sections we review how these systems and processes interact with others known to be involved in social contact and social regulation, as well as some conjectures about how (and why) the brain may be designed to minimize its own bioenergetic expenditures in maintaining desirable core body temperatures through proximity to reliable social resources. Throughout, the primary focus is on mechanisms of *increasing* body temperature, as that is the most frequently used form of thermoregulation to involve the use of social mechanisms—after all, an increase in body temperature is immediately dangerous, and a response by others would be too slow [curiously, cold (vs. warm) receptors are also found 3–10 times more likely in most areas of the body; Guyton, 1991].

#### Temperature Detection

Body temperature is set as a function of core bodily needs—such as optimal enzyme activity, the maintenance of metabolic needs, and defense against infection. Because a small window of core body temperature is necessary for optimal functioning, the brain and periphery have multiple means of predicting and detecting change both in and outside of the skin.

Humans can detect and predict temperature in many ways. The most complex of these—likely a uniquely human trait—is the ability to *predict* changes in ambient temperature long before such changes could potentially impact temperature in the body’s core. Detection of temperature can thus go via predictive systems outside of one’s own physical body. Culture affords people to use technology, and do this many days in advance—an advantage that is hard to overstate, since such advanced prediction capabilities enable people to alter behaviors and environments such that more proximal (and bioenergetically expensive) detection and adjustment measures are unnecessary.

A way related to reactive temperature control runs via complex physiological systems of temperature detection and response systems. A critical central nervous system mechanism for this appears to be the preoptic area (POA) of the anterior hypothalamus. The POA serves many integrative functions, including the utilization of multiple streams of temperature-related information sources for the purpose of both up and down regulating core body temperature as needed, and maintaining core body temperature “settings” through a variety of mechanisms. The skin is an important initial defense against changes in core body temperature resulting from changes in ambient temperatures. As with many homeostatic systems, changes in skin temperature are detectable before—sometimes long before—threats to core body temperature are detected. Although we still have limited knowledge of the precise mechanisms by which effectors within the skin relay information to the POA regarding ambient temperature, the effect is so rapid and pervasive that brain and core temperatures rarely change in response to ambient cooling of the skin (Guyton, 1991).

What is known is that cutaneous sensory information about ambient temperature is mediated through the dorsal horn of the spinal column and the lateral parabrachial nucleus of the midbrain—mechanisms that also deliver temperature information from the viscera, though precisely how this is accomplished is not as well known. Interestingly, the POA is capable of detecting changes in the temperature of circulating blood directly through a small proportion (approximately 30% for warm, less than 5% for cold) of thermosensitive neurons. These neurons are capable of altering their activity in response to even quite small changes in blood temperature and play a role in both heat retention and heat loss relevant to the brain and core (Nakayama et al., 1961; Guyton, 1991).

#### Temperature Regulation

The brain and body are capable of mounting many defenses against undesirable challenges to core body temperature. Given the diverse reactive mechanisms that are involved across the body, these strategies are often very expensive in terms of energy expenditure. Once temperature changes are detected by any or all of the means described above, regulatory mechanisms are active almost immediately. Again, central to these regulatory mechanisms is that they are hierarchically organized in terms of their relative bioenergetic costs, following from this general principle referred to as the “economy of action” (Proffitt, 2006; Davies et al., 2012).

The first and probably least expensive strategy to regulate temperature for humans involves the strategies we have alluded to above: Prior predictions and contingency planning—in short, predictive ambient temperature control with technology (heating and cooling systems) or by using thermoregulatory clothing (coats, shorts, etc.). Like other ways of outsourcing mental functions to the environment, abilities to prepare for predicted changes in ambient temperature derive from a mechanism altogether different from the mechanisms related to more proximal thermoregulatory adjustments, including prefrontal and cingulate mechanisms related to vigilance, working memory, and executive control. By being able to predict temperature in advance, it precludes more downstream thermoregulatory efforts. Being able to predict temperature in advance allows the organism to schedule energy expenditure in service of thermoregulation more efficiently. It is also exactly in the management of this costly ability that we will later locate social thermoregulatory strategies.

At the proximal thermoregulatory level, one of the first reactive mechanisms recruited to maintain optimal levels of core body temperature is also the one most associated with *predictive* temperature outcomes—the modulation of blood to the periphery of the body. On the one hand, when ambient temperatures are very high, *vasodilation* can allow more blood out to the periphery, on the assumption that there is little danger that circulating blood will adjust internal temperature. On the other hand, *vasoconstriction* is an early defensive against ambient chill, such that smaller amounts of blood are delivered to the periphery, where cooling might threaten core temperatures (though, in extreme cases of cold, vasodilation has been observed; Daanen, 2003, 2009). Importantly, this is thought to be implemented in a predictive fashion, such that cutaneous effectors within the skin detecting changes in ambient temperature cause vasoconstriction before—often long before—any such changes are detectable in the body’s core.

Often consonant with vasoconstriction strategies is *piloerection*, otherwise commonly known as “goosebumps.” Piloerection refers to the constriction of cutaneous muscles that cause hairs in the skin to stand erect. Importantly, it also causes the skin to “bunch” such that it is less porous with prevailing ambient conditions, thus conserving body heat. Importantly, both vasoconstriction and piloerection are sensitive to psychological as well as ambient temperature conditions. For example, during threat responding piloerection may serve as part of a warning signal to conspecifics (a warning that is obviously vestigial in humans), and vasoconstriction may buffer the impact of injuries by limiting potential blood loss during conflict.

By the time changes in temperature are detectible in the core (and indeed in the POA directly via thermosensitive neurons), the thermoregulatory strategies outlined above can be considered insufficient, and the body is thus forced to take reactive measures that are more bioenergetically expensive. Among the options at this stage are thermogenesis via increased metabolic activity within BAT, sympathetically mediated increases in heart rate, and shivering—the rapid, small muscle movements in and around the visceral core that increase local body temperature (Golozoubova et al., 2006).

Thermogenesis by BAT activity is fairly well characterized, especially in rodents, as a regulation mechanism for cold-induced thermogenesis (Saito, 2013). In humans, most of what is known about the vital thermoregulatory role of BAT concerns early infancy, and only recently we know that BAT plays an important role in adult humans (Nedergaard et al., 2007; Cypess et al., 2009; Virtanen et al., 2009). Remarkably, BAT recruitability in humans has even been linked to incidental cold exposures (Lee et al., 2014)^6^. Unsurprisingly, thermoregulatory activity within BAT is mediated in part through the POA, but also through circuits within the medulla and spinal column. Metabolic activity within BAT is higher than in many bodily tissues, and one consequence of metabolism is the generation of heat as chemical bonds are broken down. The POA plays a similar role in activating sympathetically mediated cardiac thermogenesis, particularly through increases in heart rate—increases that also impact overall metabolism and, hence, body heat. Indeed, increases in heart rate are mediated through many mechanisms similar or identical to those mediating BAT thermogenesis (Astrup et al., 1985), and can probably be considered alongside BAT in terms of bioenergetic cost.

By contrast—and in line with our earlier thoughts on how energetically expensive solitary thermoregulation actually is—shivering can in many ways be seen as the body’s last resort for reactive temperature homeostasis. Experimental support suggests the thermal threshold needed for shivering to commence is lower than that of any other known thermoregulatory mechanism (Badjatia et al., 2008), as heat production can be increased two- to threefold, and from an energy perspective, its efficiency is only about 10% (Yousef, 1987; Höppe, 1993). In shivering, motor neurons are rapidly fired in small rhythmic bursts that actually leave the body shaking and that generate a great deal of heat through increases in local metabolism. Indeed, by the time the body has begun shivering, there likely have been noticeable changes in core body temperature, at least at the level of thermosensitive neurons in the POA and elsewhere. Although effective, shivering is a very costly strategy, and thus best avoided if possible. Importantly, the precise mechanisms by which shivering is initiated as a defensive thermoregulatory response remain largely unknown, but are likely to include the POA, the parabrachial nucleus, and circuits of the medulla—all systems and circuits implicated in other thermoregulatory efforts.^7^

## Social Thermoregulation Saves Energy: Thermoregulation across Relationships and Development

Thermoregulation to warm the body is a costly business for many solitary animals, in part because it is tied to reactive control, but also because of the many regulatory mechanisms involved across the body. This may be best exemplified through understanding energy regulation in the case of temperature, glucose availability, and torpor (which manifests in decreased activity and lower temperature). For example, the onset of torpor is tied to glucose availability in deer mice (Nestler, 1991; Stamper and Dark, 1997). Furthermore, daily torpor has been suggested to be common in sunny regions for the marsupial *Sminthopsis macroura*, suggestively because the environmental temperature helps the animal to conserve energy (Geiser and Drury, 2003). Comparably, Sockeye salmon have been suggested to initiate torpor more frequently when food resources are limited (Brett, 1971; Lillywhite et al., 1973). Keeping all this in mind, the most efficient way to conserve energy is by seeking an optimal environment in terms of energy savings before changes in temperature occur, in which the organism can attain “thermoneutrality,” which all depends on an idiosyncratic level of comfort for the animal. In other words, the level of comfort relies on metabolic activity (Terrien et al., 2011), which differs across organisms, across the lifespan, and across social contexts.^8^ One of the key principles throughout our hierarchy of thermoregulation is that the regulation of body temperature is closely tied to limiting energy expenditure, and thus, solitary thermoregulatory activity is best minimized if possible, or, at the least, made as efficient as possible.

Thus, solitary thermoregulation is very costly and it would be cost efficient if the body had *cheaper* mechanisms in order to stay alive. Evolution has resolved this predicament through another level in our hierarchy of thermoregulation, that is, the co-regulation of body temperature through conspecifics, which allows formation of models for predictive control of the temperature environment. Interestingly enough, in human brain mechanisms for social interaction (like sex) are largely overlapping with those of behavioral thermoregulation (Satinoff, 1982). Behaviorally, conspecifics help upregulate temperature to thermoneutrality or homeostasis through huddling, and this strategy has been detected across a variety of animals.^9^ This principle is best illustrated by the social behavioral strategies that, across species, have evolved to reduce energetic costs to warm one’s body (all resulting in a reduction of the net costs of total thermoregulation for groups of animals). As one example, research reveals that African Four-Striped Grass mice utilize less energy per individual in large groups as compared to smaller groups when they are below their thermoneutral zones in the laboratory (Scantlebury et al., 2006).^10^

Beyond these direct empirical examples, a review considering eight hypotheses on the evolutionary causes for group living amongst rodents found that social thermoregulation was considered as one of only two with considerable empirical support (reducing risk of predation was the other one; Ebensperger, 2001). Ebensperger (2001) cites two types of research findings in support of the social thermoregulation hypothesis. First, sociality and communal nesting amongst species prevails for those in relatively colder areas or seasons (e.g., grouping and burrow sharing). Second, energy expenditure amongst rodents decreases if they are allowed to huddle in experiments with their conspecifics. A final compelling experimental example has been provided amongst the *Octodon degus* (a Chilean rodent), whose basic metabolic rate at 15°C is 40% lower when housed with three or five vs. alone, while the surface temperature of the huddled animal significantly increased (Nuñez-Villegas et al., 2014). Together this supports the idea that huddling causes energetic demands to be reduced, providing a co-regulation of body temperature (see also, Akin, 2011), in part through the reduction of the area of the animals that is exposed to the environment. We will first address this strategy in the following section. Later we also discuss thermoregulation throughout development and caregiving, and apply reactive and predictive control to forming predictive models of social thermoregulation from early infancy to later life.

That thermoregulation is done socially can be found across homeotherms and poikilotherms, and research observations have revealed that close kin do so to support the weakest in the group, both throughout development as well as incidentally: Homeotherms for example engage in longer lasting social thermoregulation like nest-sharing to increase the chance of survival for infants. Further, many animals also do so reciprocally, as adult birds and mammals shield each other from harsh climatic environments through huddling (Gilbert et al., 2007; Terrien et al., 2011). We now turn to examples from the animal literature where huddling as a means for social thermoregulation has been detected.

### Communal Ways to Thermoregulate Socially

The strategy of huddling has been detected across different rodent families. The overview by Ebensperger (2001) shows that huddling strategies in the service of energy conservation has been detected across many different rodent species.^11^ In humans, there may be comparable strategies to reduce the energetic costs of thermoregulation. Most of the research efforts in humans on huddling (which in humans focuses on touch) to date however have been dedicated to other (related) areas of research, such as social judgment (Erceau and Guéguen, 2007), prosocial behaviors (Crusco and Wetzel, 1984; Hornik and Ellis, 1988; Field, 2010), valence of affect (Vallbo and Johansson, 1984), and emotional communication (Hertenstein et al., 2006, 2009; for reviews, see Gallace and Spence, 2010; Tjew-A-Sin and Koole, 2013).

Yet, surprisingly little research has been devoted to the role of touch, emotion regulation, and the basic metabolic rate amongst humans. One study has linked face and body temperature specifically to the role of touch in sexual arousal, where skin temperature of the face increased after highly intimate (face and chest) contact (Hahn et al., 2012). There are some other studies that were documented on the role of skin-to-skin contact and its relation to skin temperature which may show the connection between touch and skin temperature, to which we return when we discuss developmental thermoregulation. More indirect links have been provided between close relationships and the regulation of metabolic resources: Henriksen et al. (2014) found that being socially integrated is related to a lesser consumption of drinks containing sugar during pregnancy, while thinking about a romantic partner (who is likely to be a source of warmth) leads to a slight increase in glucose levels (Stanton et al., 2014). The first part of a research agenda on social thermoregulation should thus focus on uncovering the relationship between social integration, thermoregulation, and metabolism regulation in humans.

To close this section, the supposed link between social thermoregulation and energy conservation is not without exception: The support for energy conservation as dependent on seasonal changes in ambient temperature through group formation was not detected in prairie voles *Microtus ochrogaster* (Getz et al., 1993; Getz and McGuire, 1997; but see Getz and Hofmann, 1986; Getz et al., 1987), or seems unrelated to seasonal changes in ambient temperature (Blumstein and Arnold, 1998). We think of these latter (rare) findings as outliers in the role of social thermoregulation in the service of energy conservation, and potentially helpful in understanding the boundary conditions of social thermoregulation.

### Thermoregulation Throughout Development

The first research example on social thermoregulation that is well known to psychologists is that of Harry Harlow’s rhesus monkeys. In his most famous contribution on this topic, *The Nature of Love*, Harlow (1958) discussed rhesus monkeys that he raised either with a surrogate mother made out of wire, or one made out of terrycloth (the surrogate, non-soft wire mother caused greater psychological problems in the rhesus infants’ later lives). In a lesser-known contribution, Harlow and Suomi (1970) discussed their observation of a rhesus monkey raised with a physically warm surrogate mother. After switching to a physically cold surrogate mother, the researchers noted that the infant rhesus monkey displayed remarkably less affiliative behaviors with this cold surrogate mother, and was also much less explorative. Once Harlow and Suomi (1970) switched back the warm mother, the rhesus monkey quickly returned to the original level of affiliative and explorative behavior and “forgave” the surrogate for her coldness. Conversely, for the infant rhesus monkey that was originally raised with a cold surrogate mother the level of affiliative behavior never reached that level of the rhesus monkey originally raised with a warm mother displayed.

#### Thermoregulating the Young

The anecdote by Harlow and Suomi (1970) provides fascinating directions to explore how thermoregulation functions in early development. One of the ways to explain their observation may well be that one of the most stressful and risky periods that animals have in their lives is just after being born and the period thereafter. And, in fact, the security that a warm feeling offers against cold stress should help regulating the infant, mostly to be protected from hyperthermia. The length that an infant needs to be thermoregulated by a caregiver in its quest to survive varies species-by-species. As one example, golden hamsters are able to regulate their own body temperatures by the age of 14 days (Leonard, 1982). Human infants are even more fragile and need to rely on older individuals for a considerable time for the regulation of body temperature to prevent falling prey to reactive thermoregulation (Winberg, 2005).

In general, the huddling strategies that we have discussed in the previous section also prove their use in the care of human neonates. Infants across many animal species do not yet have the surface to body area to be able to thermoregulate their own body. Young infants have a stress hyporesponsive period (SHRP), and maternal thermoregulation maintains this SHRP, protecting the developing brain of the infant (at least in rats), further allowing it to mature for predictive capacity (Suchecki et al., 1993). On the flipside, maternal separation (and the inevitable coldness and unpredictability of the environment) leads to elevated stress levels. For a human infant these elevated stress levels immediately pan out in a greater deviation between core and skin temperature, and this thermoregulatory risky state can even lead to the infant’s death (Mori et al., 2010). Thermoregulation by the caregiver is required for survival and we thus suspect that the same process of brain maturation due to maternal thermoregulation also occurs in humans.

Additionally, for altricial mammals, episodes of hypothermia have been suggested to reduce growth through a reduction of biochemical activities. Again, the reasons for instability in thermoregulation and a following insufficient growth are likely to be found in the problem of energy expenditure. Mothers have typically “solved” this problem by having a body that is evolved to deal with this. For example, the neuromodulator oxytocin is typically involved in nursing behaviors, such as touching and breastfeeding. Additionally, energy intake (at least in mother rats) can be increased through increased hunger that is related to the secretion of oxytocin. Further support for this idea is that in caregiving rats, if the neural pathways for milk ejection are lesioned, energy intake is decreased back to their normal levels (Uvnäs-Moberg and Eriksson, 2008).

Harlow’s studies on rhesus monkeys showed how the infants had a preference for warm surrogate mothers, or cotton cloth mothers that provided physical comfort. The explanation for these studies—in line with our reasoning—is that the warm, comfortable mother provided metabolism regulation. Comparably, rat dams choose an environment (a warm one) in which it is less costly in terms of thermoregulation to provide their caregiving. Reminiscent of Harlow’s studies, this *even* occurs when their nest is in a colder environment (i.e., they prefer warmer environments over their own nests). Interestingly enough, these rats were observed to optimize their expenditure of resources: When they could choose their thermal environment, the rat dams chose environments that were somewhat below their thermoneutral zone. They did so because typically require less feeding in somewhat colder environments. The author argued that this helped the rat dam to conserve energy (see, McFarland, 1977). Together, this thus further supports the idea that that thermoregulation is closely tied to being as energy efficient as possible.

Just like incidental forms of thermoregulation (e.g., to shield from harsh environments), developmental thermoregulation is typically achieved through huddling. For example, Norway rats follow economy of action principles in caregiving through forming a huddle, which decreases the exposed surface area of the developing pups relative to their internal heat producing system (Woodside et al., 1981). Shielding young animals from harsh environments is very important in the context of reproduction, which is why many species use this strategy to protect their young. In marmots, juveniles profit most from huddling as they typically have the lowest fat reserves (Armitage et al., 1976; Arnold, 1988). Also for infants of emperor penguins, a species that assumes collective breeding tasks during extremely cold Antarctic winters, huddling increases the chances of survival (Gilbert et al., 2007).

#### Risk for Caregivers in Provision of Social Thermoregulation

This kind of thermoregulation is rightfully communal, as it is not without risk for the caregiver: Taking care of the infant’s body temperature increases the chances for disturbances in the caregiver’s thermoregulation processes. Lactating Norway rats for example are vulnerable to acute hyperthermia when in contact with their pups (Woodside et al., 1981; Adels and Leon, 1986). Furthermore, loss of mass is greater and survivorship is lower for marmots that have to care for their own infants (without the help of subordinate marmots), while also the loss of mass for both the caregivers and subordinate marmots is greater with the presence of infants (Armitage, 1999). Although in humans these severe risks seem less likely because of the way our cultures have evolved, these findings for animals suggest that social thermoregulation first evolved in the context of Communal Sharing relationships (= in the context of close kin, providing for the other, even at the risk of death for the self). Still, while there may be no immediate risk of death for humans, such *communal thermoregulation* for humans is still heavy in terms of energy demands, and should mostly occur with predictable relationship partners (a finding for which we have some preliminary support from our own lab; Wagemans et al., 2014).

#### Evidence in Humans of Development Thermoregulation

There are some indications that skin-to-skin contact closely relates to the maintenance of body temperature. One research example revealing the importance of skin-to-skin contact finds that feet of babies that were held skin-to-skin, as compared to those that were removed from the skin of the mother (and swaddled in six cotton cloths and a cotton blanket as an outer layer) revealed a greater increase in skin temperature. Beyond that this provides first support for the co-regulation of body temperature throughout development, the researchers proceeded to conclude that young infants are equipped with some kind of innate program to seek warm comfort from their caregiver (Bystrova et al., 2007; see also Caporael, 1997; Jonas et al., 2007; IJzerman et al., 2012).

After reviewing such experimental studies on skin-to-skin contact, Winberg (2005) suggested that keeping the infant against the skin has major effects on the feelings of security of the infant, with infants crying less and having a smaller reduction in skin temperatures. For example, children that were just born showed differences in skin temperatures depending on treatment condition. Children were swaddled in six layers of cotton cloth, placed in the mothers’ arms, or held skin-to-skin with the mother. The decrease in skin temperature, which accompanied an increase in crying and interpreted by the authors as being a result of the “stress of being born,” was greatest in the swaddling condition, then the condition in which newborns were placed in their mothers’ arms, and the smallest temperature decrease was found for the newborns that were held skin-to-skin with their mother (Bystrova et al., 2003).

We can reconsider earlier research conducted by one of our own labs (IJzerman et al., 2013) and other labs (Fay and Maner, 2012) in light of our theory. In both studies, people’s perceptions of the reliability of their social world (their attachment styles) moderated the effects of warm (vs. cold) cues. This is important, because the review by Winberg (2005) further suggested—but provided little empirical evidence for—the idea that skin-to-skin contact in the infants’ early life stages also contributed to a better relationship with the caregiver (and potentially to later social relationships). The research examples we cite provide first such evidence for the hypothesis Winberg (2005) proposed. While early research support thus exists, we add as an agenda point the importance of testing the increased predictability of energy provision through social thermoregulation.

## Consequences for Psychological Mechanisms

Most of what we have provided until here can be explained through people’s direct interaction with the environment, and much of it can be understood through a direct coupling of action and agents that can mostly rely on biosocial models (e.g., Chemero, 2009; Beckes et al., 2014). But we would like to extend our model further, and provide a first consideration into what our ideas may mean for more complex cognitive systems. We think that the consequences are twofold: First, and most obvious to psychologists, there should be considerable consequences of including our model of social thermoregulation into a predictive model of self and others (i.e., attachment style). We will reinterpret existing research and discuss the formation of self, and the formation of the predictive model for social thermoregulation. Furthermore, we discuss earlier research in light of our theory, and indicate how people use temperature estimates for prospection. Finally, our theory should also more closely tie such predictive models to basic physiological mechanisms, and these should in turn be heavily influenced by their social context.

### Social Thermoregulation and the Development of Higher Order Cognition

The development of higher order cognition makes it possible for human beings to live the way they do. Higher order cognitive functions, like predictive analysis, inhibitory control, self-reflective consciousness, abstract thinking, willed action, and a theory of mind are on top of the hierarchical structure and typically are thought to correlate with activity in the prefrontal cortex (e.g., Dietrich, 2003). These types of activities allow people to participate in complex cultural structures (Baumeister, 2005), partake in complex social normative systems, and choose environments that support these abilities (Lindenberg, 2013).

We believe that effective social thermoregulation is one crucial facet that makes this all possible, by allowing growth of the prefrontal cortex combined with the development of internal models of one’s social environments. The growth of these higher order structures—metabolically speaking—is expensive. We think that the infant brain makes predictions about the availability of energy in early life. When the caregiver is available for reliable thermoregulation, it allows for dedication of the excess energy for the maturation of the infant brain, which should considerably impact the growth of structures related to higher order cognitive functioning (Carter, 2014).

Indeed, as we noted before, maternal thermoregulation protects the developing brain of the infant (at least in rats), allowing it to mature for greater predictive capacity. Because harsh environments *may* stimulate BAT growth, the bioenergetic resources typically devoted to thermoregulation could potentially be invested into growth of other areas of the brain that allow for higher order control.^12^ In turn, we predict that adverse early social experiences will affect these *sociophysiological mechanisms*.

And there is indeed some evidence that the degree to which people are thermoregulated in their early days shapes higher order cognitive functions, like levels of self and/or executive control. Attachment security—which is typically related to predictive control—correlates with enhanced self-regulation (e.g., Mikulincer et al., 1993; Mikulincer and Florian, 1995), and also with a more coherent self and with greater self-complexity (Mikulincer, 1995). We interpret this evidence tentatively as meaning that the “self” and its regulation emerges from the interaction with and being regulated by close others (see for comparable reasonings Häfner and IJzerman, 2011; and also Righetti et al., 2013), and that one of the earliest mechanisms of these is through social thermoregulation.

The hypothesis that emerges then that greater skin-to-skin contact (like the examples we cited earlier) should stimulate people’s capacities to self-regulate. This has been found: regular skin-to-skin contact in early life improves executive functioning for a child (Feldman et al., 2014). While such early research support exists, our ideas are still quite tentative, and the research agenda should now focus on establishing (1) more direct links between social thermoregulation and the development of higher order cognitive functions, (2) whether responsive thermoregulation to the infant’s stress improves self-control/executive functioning in later life (mediated by improved BAT functioning and greater growth in predictive systems), and comparatively whether (3) animals that engage in “more effective” thermoregulation, also have greater brain mass related to predictive functioning (see, e.g., Dunbar, 1998).

### Forming Predictive Models of Relationships—Attachments

One of the hallmark features of attachment theory has been the concept of the predictive model of others (e.g., Craik, 1943; Bowlby, 1969; Hazan and Shaver, 1987; Mikulincer and Shaver, 2003; but see also Beckes et al., 2014). In the recent past, several notions that we consider central to predictive models have been discussed, viz., conceptual metaphor theory (Lakoff and Johnson, 1999), neural reuse theory (Anderson, 2010), and perceptual symbol systems (Barsalou, 1999, 2008). We build on perceptual symbol systems and neural reuse theory here (for explanations for our preference, see IJzerman and Koole, 2011; Beckes et al., 2014). Building on these theories and Tops et al.’s (2010) theory of Predictive and Reactive Controls Systems, we explicate a general reliance on predictive control to understand how we form models of our social world. The discussion of this support may also help understand why we think that thermoregulation is causally involved in the formation of the internal model.

#### Evolved Simulators: Innate Expectations

Others have argued that people are born with systems that have evolved throughout different generations through repeated assemblies (Caporael, 1997). Because of the importance of procreating and giving warm care, infants should have “evolved simulators” to seek warm contact in early life stages that provides an environment that is predictable for their regulation of their metabolic resources (see also Barsalou, 1999, 2008; IJzerman et al., 2012).^13^

There is some support for the idea that if infants do not meet “warm” experiences, they will face negative consequences. In a relatively controversial experimental study, Bystrova et al. (2009) found that early separation of the infant from the skin of the mother led to poorer self-regulation in the infant 1 year thereafter, very suggestively a sign that the separation of the warm skin of the caregiver altered their predictive models of the world. And, indeed, some early support exists that the infant has connected their predictive model (i.e., attachment styles) with temperature cues (Fay and Maner, 2012; IJzerman et al., 2013). Attachment has been directly linked to social thermoregulation in relation to attachment and predictability as well: Infants ranging in age from eight to 16 weeks showed drops in skin temperature in response to their attachment figure (the mother) leaving the room (the typical set up in the famous Strange Situation paradigm; e.g., Ainsworth and Bell, 1970), and the presence of a non-attachment figure (a stranger) without the attachment figure caused the same effect. Importantly, these effects were solely detectable in skin temperature, but not in differential responses in smiling or crying (Mizukami et al., 1990; see also Mizukami et al., 1987). There is thus some early support for linking attachment and affiliation to thermoregulation. An important point on the research agenda for social thermoregulation is thus to find out whether infants are predisposed to seek for specific thermo-related cues, and whether differences in contingent thermoregulation from the mother to the child leads to differences in attachment styles.

#### Formation of the Predictive Model

In further elucidating this developmental link, we need to understand the organization of the predictive model, which is likely to rely on (at least) two factors, one that is reliant on basic physiological development and another that is part of the organism’s cognitive development (a point we already discussed above). We think that early social development contributes to the way the organism’s body develops later on, and one important facet of this development should entail the earlier discussed BAT (see Cannon and Nedergaard, 2004). The link between oxytocin secretion and touch is well known (e.g., Uvnäs-Moberg et al., 2015). Curiously, deficiencies in the oxytocin receptor gene have been associated with problems in the development of BAT in mice (Takayanagi et al., 2008). We thus think that BAT, seen as important for thermogenesis, could even play a role in determining meaningful psychological individual difference patterns, and that a place to examine this is in individual difference patterns of people’s thermoneutral zones (Kingma et al., 2012, 2014).

We believe that BAT may develop in different ways in case of early aversive postnatal experiences—through ways that are still to be explored. Understanding the development of BAT will be important for also further understanding attachment, as the absence of reliable and physically warm caregiving may even lead to changes in BAT development, affecting social thermoregulation abilities and even the range within which the predictive model can operate.^14^ In other words, some effects (and specifically—though not only—of temperature cues) that previously have been attributed to predictive models (i.e., attachment styles) alone may be due to differential physiological factors—and this should be part of a research agenda for social thermoregulation. And for interpersonal relationships a predictive model is more complex still. A predictive model for relationships should rely on what has been previously coined as *convergence zones* to form coherent representations of the social world.^15^ Convergence zones for close relationships are likely formed through repeated caregiving behaviors (like breastfeeding), which are likely to contribute to predictive models through the activation of opioid and oxytocin systems (Pedersen and Prange, 1979; Uvnäs-Moberg, 1996; Winberg, 2005), and we think that these convergence zones allow people to run simulations on what their social world will be like, by merely thinking about caregiving, having sex, or about being excluded.

But how can we know that thermoregulation is causally involved with the internal model? In social psychology, such questions are typically resolved through priming methods, which is the idea that people utilize cognitions that are reliant on the activation of recent other stimuli (Neely, 1977; Tulving and Schacter, 1990; Bargh and Chartrand, 1999; Bargh et al., 2012). And indeed, a warm cup makes people judge others as more sociable, and makes themselves more generous (Williams and Bargh, 2008), while warmth (vs. cold) also makes people perceive more relationships in their environment and construe an experimenter as more overlapping with the self, and makes people more likely to use more relational language, be more cooperative, and trust others more (IJzerman and Semin, 2009; Kang et al., 2011; Storey, 2013; Schilder et al., 2014).

There is thus support that social thermoregulation is at least involved in the internal model of the close relationship. But that does not mean that social thermoregulation is the *only* factor in forming predictive models of relationships. Going back to Damasio’s (1989) idea, convergence zones are “uninformed as to the content of the representations they assist in attempting to construct” (p. 46), and thus rely on the multiple—and bi-directional—inputs across body and brain that are crucial for predictive models of relationships. But what is the content of internal models that are formed on the basis of this process? And how should they update? We think they are likely slow in updating, and based on what we have reasoned there should be an important role for thermal cues (the content of our knowledge about relationships) through reactive control systems into predictive control systems—including the predictive model that provides us with a report of the social “weather,” which can both be longer term (predictive) or help us respond in the here and now (reactive). In making our model even more detailed, we further explain how Tops et al.’s (2010, 2014a,b) theory of PARCS can explain some of the earlier evidence that has been collected.

### The Further Formation of the Predictive Model

Predictive and reactive control systems specifies two levels of control—one predictive and one reactive—and the theory allows for a way to integrate thermal cues into broader representations, which are formed through the slow updating of information regarding one’s social world. From this perspective, motivational control can be shifted between predictive systems and reactive systems. Broadly speaking, this suggests that reactive control systems evolved early in evolutionary history for the purpose of behavioral control in unpredictable environments, like harsh climates^16^. The reactive control system is thought to specialize in the processing of novelty (cf. Whalen, 2007), biological salience (cf. Adolphs, 2010), and urgent environmental stimuli in order to react to exigencies, such as is the case for newborn infants and their (thermoregulatory) expectations of their social world. The reactive system functions in a feedback-guided manner to the immediate situation and focuses attention narrowly on the local situation. In this manner it can take new information—whether the parent will regulate their temperature or not—and is able to communicate with the predictive systems to update predictive models promoting greater predictive control in the future (Hasher and Zacks, 1979; Tops et al., 2014a,b).

The reactive system helps guide people to gage and act on their momentary resources [that are specified by people’s gage for (social) warmth vs. coldness], due to an integration of early perceptual cues into the predictive models. These should—at least situationally—pan out in “working models of relationships” (Craik, 1943; Bowlby, 1969). Repeated patterns should lead to a slow updating of such a model. Earlier we have referred to the formation of higher order cognitive functions as a result of maternal thermoregulation. The brain areas associated with these higher order cognitions (of which the predictive system is a part) are thus believed to largely be an outgrowth of evolutionary pressures that emerged in highly predictable and stable environments (Tops et al., 2014a,b). The predictive system supports a variety of cognitive functions that are representational and semantic in nature, one of the most important of which is related to thermoregulation, because it is so essential to many animals’ survival.

The predictive system is further involved in cognitive tasks with internally focused attention such as imagining a different time or space (Buckner and Carroll, 2007), or another person’s perspective (Waytz and Mitchell, 2011). Craik (1943) suggested that using predictive models allows for testing alternative possibilities, and making better predictions regarding situational outcomes. In the same sense, PARCS suggests that the predictive system’s function is to run simulations to predict future events. Thus, the predictive system engages in creating internal models that predict future outcomes through simulations, and updates those models slowly, in line with the idea that it responds to environmental predictability. Together, this means that the predictive system helps people in better scheduling metabolism, and thus better using the “weather report” they have obtained of their social environment. The way that perceptual simulations thus are likely to function is by calling on the relevant information from named convergence zones, which would include relevant features of the entire thermoregulatory state of the processes we have discussed above (for how simulations work, see Barsalou, 1999, 2008).^17^

### Temperature Estimates and the Regulation of Metabolic Resources

To what degree then is PARCS supported with regard to social thermoregulation? If indeed the reactive system helps guide the agent through gaging momentary resources, then one should see differences in estimation of ambient temperature in relation to social resources, and second, there should be a link between thermoregulation and the regulation of social behavior. The ability to gage resources could be something that we have referred earlier to as the “weather report.” And indeed, there are a number of reports that show the link between the “weather report” (i.e., the gage of resources) and close relations: Primes of social/physical similarity (vs. social/physical distance and exclusion) lead people to estimate temperature as higher, whether this is about the relationship (Zhong and Leonardelli, 2008; IJzerman and Semin, 2010), the self/other (Szymkow et al., 2013), or consumer products (IJzerman et al., 2014). IJzerman et al. (2014) even find that temperature cues are causally implicated in the willingness to purchase the consumer products that make them feel warmer (for comparable findings, see Van Acker et al., 2015).

These findings should tell us that the participants perceive the social world to be filled (or not) with resources, and that higher (lower) estimates of temperature may suggest to them that their world is socially more (less) predictable. And people can act on momentary resources, and self-regulate their own feelings that relate to a lack of being close to others: Holding a warm object has been found to alleviate the detrimental effects associated with brief social exclusion (Bargh and Shalev, 2012; IJzerman et al., 2012).^18^ The direct experience of physical coldness has also been found to lead to a desire to be with others (vs. being alone) and an increased preference for romance movies, and—crucially—the latter only occurred for people associating these movies with psychological warmth (Hong and Sun, 2012; Lee et al., 2013). In addition, among PTSD patients, loneliness has been found to positively relate to a preference for warm foods (Li and Liao, 2013). Finally, in colder conditions people self-regulate through socially warm experiences: Nostalgia is triggered by coldness (Zhou et al., 2012; Sedikides et al., 2014), people seek socially warm experiences when they are cold (Zhang and Risen, 2014), and they judge houses to be more homely when they are colder (Van Acker et al., 2015; for a review, see Raison et al., 2015). There is even some preliminary support that skin temperature is responsive to someone else’s stress (Vuorenkoski et al., 1969; Wagemans et al., 2014), which we think serves in social emotion regulation. It is still crucial however to show that skin temperature of a supporter is causally involved with the regulation of another’s emotion.

We regard this wide array of findings as supportive of our model. Nevertheless, whether these mechanisms lead to the *development* of stable individual differences remains an informed conjecture at this stage and we also add this to the social thermoregulation research agenda. First, of course, there is the potential for differences in growth of the infant brain, and having greater control over reactive cues (vs. predictive cues) may be adaptive for the individual organism in unpredictive environments. Second, it could be that the development of BAT goes hand in hand with expectations of one’s social network. Specifically, deficiencies in the oxytocin gene receptor have been implicated in poor development of BAT in mice (Takayanagi et al., 2008), and if mice are poorly thermoregulated, they develop more BAT (Heldmaier, 1975). Basically, it could be that unreliable warmth-related caregiving in the infant’s early life contributes to impaired development of BAT, and therefore also to a predictive system that relies on a different physiology.

## In Closing

We have made what we believe to be a first foray into what we think is extremely important to human functioning: Social thermoregulation. In so doing, we have described a kind of temperature homeostasis that relies on predictive and reactive control systems, from behavioral thermoregulation to the formation of predictive models of self and others. We have also noted quite a number of empirical findings that are supportive of our model, but realize that much remains to be explored. Nevertheless, we think that our theory of social thermoregulation will achieve broad applicability across domains in psychology. For example, to what degree are findings that we detect in attachment studies due to predictive models, and to what degree do they rely on the development of BAT? Do the predictive models for social thermoregulation also play a role in other types of relational models than communal sharing (see, e.g., Fiske, 1992)? How does social thermoregulation relate to findings that have been typically implicated in the domain of self-regulation, like obesity? And how does sexual behavior in humans relate to social thermoregulation, and how can it aid vs. impede the formation of communal bonds? Finally, if social interactions are at least in part predicated upon social thermoregulation, then do novel technological developments that help humans resolve energy regulation decrease a direct need for (face-to-face?) interpersonal relationships? At any rate, we hope that our theory will become truly generative, and that it will help the research community to explore many of these questions.

### Conflict of Interest Statement

The authors declare that the research was conducted in the absence of any commercial or financial relationships that could be construed as a potential conflict of interest.

## Footnotes

1 Thermoneutrality, or the thermal neutral zone, is the temperature at which the organism does not need to regulate its body temperature (in near naked male human subjects this is between 33°C and 35°C; Savage and Brengelmann, 1996).

2 It is not impossible that social thermoregulation is implicated in other relational models, like Authority Ranking. In fact, whether marmots take greater risks in providing warmth to others is partly related to their status in the group, with subordinate marmots needing to provide greater warmth by being on the outside of the group (Armitage, 1999). Similarly so, thermoregulation may even take place in Equality Matching relationships (for example when people meet a potential romantic interest for the first time, which then may move into Communal Sharing; cf. Clark and Mills, 1993). However, we think that, evolutionarily speaking, social thermoregulation primarily developed in the communal sharing context. Social thermoregulation allows for direct survival, and it would have thus been very beneficial to share metabolic resources “to be relatively kind and altruistic to people of [one’s] own kind” (Fiske, 1992, p. 691).

3 When we allude to predictive models in this paper, we refer to a predictive control system (as opposed to a reactive control system) that guides behaviors through internal models of the environment, which are formed in long-term memory and kept stable by slow learning of the environment’s predictability. The idea of the internal model originated with Craik (1943) and was later applied by Bowlby (1969), and the ability to apply the internal model to predict future events was noted by Craik (1943) to be an adaptive property of human thought. This idea of the internal model has recently been applied to so-called “situation models” (Zwaan and Radvansky, 1998), and its application in predictive control systems relies on brain networks like the default mode network (that includes the medial prefrontal cortex, posterior cingulate cortex and precuneus) and the dorsal executive network (that includes the dorsolateral prefrontal cortex; see Tops et al., 2014b).

4 Despite its centrality in the early life of the infant, we clearly do not intend to say that social thermoregulation is the *only* feature that is crucial to early attachment, as other “perceptual building bricks” are clearly also important to the life of the infant (like smell, moving in synchrony, and responsive interactions by the caregiver).

5 There is an additional kind of thermoregulation, called kleptothermy. Kleptotherms are organisms that regulate their own body temperature by stealing heat from other organisms. Kleptothermy mostly happens in poikilotherms and is unilateral, but also happens to some degree in homeotherms. In homeotherms, kleptothermy is mostly reciprocal (Brischoux et al., 2009). We do think that kleptothermy is an interesting class of thermoregulation, potentially in relation to communal sharing relationships. However, the complexity of this topic means that it deserves an entirely separate discussion, which is why we do not further elaborate on it here.

6 Interestingly enough, Lee et al. (2014) suggest that the change in BAT radio density may well be due to a process they refer to as “fat-browning” or the transition of white adipose tissue into brown adipose tissue. Based on this, we can ask the question to what degree social factors, such as the predictability of one’s social environment, play a role in fat-browning, and to what degree BAT is similarly organized in terms of predictive and reactive temperature homeostasis.

7 What we are thus not trying to say is that individual differences in thermoregulatory efforts/abilities do not occur across individuals and even across cultures. It is likely that the body has adapted to its temperature environment and to the direct social exigencies. There are clear indications that there are differences in thermoregulatory abilities across cultural/ethnic groups (Daanen, 2003; Maley et al., 2014), gender (Davies, 1979), individuals (Havenith, 2001), and lifespan (Davies, 1979; McDonald et al., 1989; Scarpace et al., 1994). To what degree these thermoregulatory differences rely on specific cultural, social, or physiological factors, remains to be seen.

8 Within the social psychological literature, it is common to revert to explanations based on positive or negative affectivity, also in relation to temperature cues (Zajonc, 1980). Beyond that this alternative process has been empirically negated (see, e.g., Szymkow et al., 2013, for one example), from the idea that we propose here it also follows that skin and ambient temperature are co-dependent and the valence of a temperature cue depends on the demand of the organism and the environment, and it is likely that preferences rely on thermoregulatory inferences.

9 We note that they “upregulate” as the cooling of the body does not nearly pose as much as a threat to the body as an increase in temperature. Downregulating body temperature through others is thus not likely to be quick enough to save the organism from sustaining brain damage.

10 One reason why net energy expenditure is lower is because being thermoregulated indicates an increased predictability of the environment and is related to activity of the vagus nerve. Specifically, a decreased predictability of the environment is associated with an increased activity of people’s reactive control systems, which is typically more costly than the activation of predictive systems. Supporting this idea, vagal tone is associated with social wellbeing and social support seeking (Geisler et al., 2013).

11 Evidence on huddling strategies was cited by Ebensperger (2001) on Bathyergidae (*Heterocephalus glaber*—Withers and Jarvis, 1980), Muridae (*Abrothrix andinus*, *Abrothrix lanosus*—Canals et al., 1997; *Apodemus agrarius*—Tertil, 1972; *Apodemus flavicollis*—Fedyk, 1971; *Clethrionomys glareolus*—Górecki, 1968; Gebczyński, 1969; Gebczyńska and Gebczyński, 1971; *Eligmodontia typus*—Canals et al., 1997; *Meriones unguiculatus*—Contreras, 1984; *Mus musculus*—Prychodko, 1958; Martin et al., 1980; Contreras, 1984; *Microtus townsendii*—Andrews et al., 1987; *Ochrotomys nuttalli*—Springer et al., 1981; *Ondatra zibethicus*—Bazin and MacArthur, 1992; *Peromyscus leucopus*—Glaser and Lustick, 1975; Vogt and Lynch, 1982; *Peromyscus maniculatus*—Andrews and Belknap, 1986; *Phyllotis darwini*—Canals et al., 1997; and *Reithrodontomys megalotis*—Pearson, 1960), Myocastoridae (*Myocastor coypus*—Moinard et al., 1992), Octodontidae (*Octodon degus*—Canals et al., 1989), and Sciuridae (*Ammospermophilus leucurus*—Karasov, 1983; *Glaucomys volans*—Stapp et al., 1991), but also for round-tailed ground squirrels *Spermophilus tereticaudus* (Dunford, 1977), southern flying squirrels *Glaucomys volans* (Stapp et al., 1991; Layne and Raymond, 1994), gray squirrels (Koprowski, 1996), house mice (Morton, 1978), meadow voles *Microtus pennsylvanicus* (Madison et al., 1984), red-backed voles *Clethrionomys rutilus* (West, 1977), white-footed mice (Nicholson, 1941), and wood mice *Apodemus sylvaticus* (Wolton, 1985) during cold parts of the year (while converging support has also been detected in deer mice, white-footed mice, and free-ranging alpine marmots *Marmota marmota*; Sealander, 1952; Andrews and Belknap, 1986; Arnold, 1988, 1990a,b).

12 Curiously enough, the development of the infant’s body to be able to thermoregulate solitarily seems to go hand in hand with the development of this specialized organ, which has been implicated in internal thermoregulation (Cannon and Nedergaard, 1985; Grigg et al., 2004; Ivanov, 2006). Indeed, in oxytocin receptor deficient mice brown adipose tissue was impaired (Takayanagi et al., 2008), tentatively suggesting that brown adipose tissue develops differently in accordance with one’s (innate) social abilities, and possibly one’s social experiences. More potently, mice that interact in larger groups (and are thus better thermoregulated) have less BAT, which suggests greater “fat-browning” in harsher environments (Heldmaier, 1975).

13 Different scholars have identified these systems with different names. Fiske (2000, p. 82) identifies these as mods. He defines mods as “an innate proclivity to coordinate social interaction in a universally structured yet culturally organized way. It is an evolved but incomplete attentional–cognitive–learning–motivational system for organizing social interaction that cannot function without a congruent cultural complement.” We are in complete agreement with his statement. However, Fiske (2000) also identifies mods to be functionally neurologically modular (which disagrees with our position on neural reuse; Anderson, 2010). Thomsen et al. (2011) and IJzerman and Cohen (2011) identify these innate proclivities as “innate input analyzers” and “pre-wired embodiments.” We use “evolved simulator” as this most closely aligns with the broader literature on the formation of predictive models.

14 There is also growing support for gender differences in brown adipose tissue thermogenesis. This suggest that this process is enhanced in females leading to differential experiences in warmth between huddling male and female mice of 8 days old and thus possible gender differences through early interaction experiences (Harshaw et al., 2014).

15 Convergence zones were first discussed both by Barsalou (1999, 2008) and Damasio (1989) as their role being “to enact formulas for the reconstitution of fragment-based momentary representations of entities or events in sensory or motor cortices” (p. 46).

16 Criticism has arisen in regards to dual process models (see, e.g., Keren and Schul, 2009). We do not see PARCS as a comparable dual process model. Indeed, Tops et al. (2014a,b) use “the terms reactive and predictive as labels to refer to functional systems that combine a set of features that together are optimal in controlling behavior and physiology in unpredictable or predictable circumstances. For example, some features of the reactive system do not fit at all to the label reactive (e.g., sustained attentional control or active maintenance of task goals by the reactive system). Moreover, each system contains certain features that are evolutionarily older and seem more primitive (e.g., related to reactivity, impulsivity, stimulus controlled) while other features of the same system are evolutionarily more recent and appear at a higher (e.g., cognitive control, reflective) level. Predictive control by internal models—and the chances of focusing one’s mental state inward—may have evolved later and at first glance appear more “sophisticated” than reactive control. However, the point we want to make is that both types of systems are continuing to evolve and have *both* developed higher-level control.” Moreover, PARCS does not describe just two systems (one being reactive and the other being predictive), nor does it only include systems that are either purely reactive or purely predictive (Tops et al., 2010, 2014a,b; Tops and Boksem, 2011).

17 As Barsalou (1999, 2008) stated, these simulations are not likely to function like a complete simulation of the original perceptual state, but are likely to be incomplete (and function much like a Gestalt), while specific goals are likely to call on activating different parts of the convergence zones we have alluded to.

18 For skepticism regarding the shower studies, see Donnellan et al. (2014) and Ferrell et al. (2014), and for skepticism of this skepticism, see Shalev and Bargh (2014).
